# Prevalence and Risk Factors of Gastric Adenoma and Gastric Cancer in Colorectal Cancer Patients

**DOI:** 10.1155/2016/2469521

**Published:** 2016-12-26

**Authors:** Dae Hyun Tak, Hee Seok Moon, Sun Hyung Kang, Jae Kyu Sung, Hyun Yong Jeong

**Affiliations:** Division of Gastroenterology, Departments of Internal Medicine, Chungnam National University School of Medicine, Daejeon, Republic of Korea

## Abstract

*Background/Aims*. To evaluate the incidence of gastric adenoma and gastric cancer in colorectal cancer patients, as well as the clinicopathological features that affect their incidence.* Methods*. Among patients who underwent surgery after being diagnosed with colorectal cancer between January 2004 and December 2013 at Chungnam National University Hospital, 142 patients who underwent follow-up upper gastrointestinal endoscopy were assigned to the patient group. The control group included 426 subjects randomly selected. The patient group was subdivided into two: one that developed gastric adenoma or cancer and one that did not. Clinicopathological characteristics were compared between these groups.* Results*. In total, 35 (24.6%) colorectal cancer patients developed a gastric adenoma or gastric cancer, which was higher than the number in the control group (20 [4.7%] patients; *p* < 0.001). Age, alcohol history, and differentiation of colorectal cancer were associated with higher risks of gastric adenoma or gastric cancer, with odds ratios of 1.062, 6.506, and 5.901, respectively.* Conclusions*. In colorectal cancer patients, screening with upper gastrointestinal endoscopy is important, even if no lesions are noted in the upper gastrointestinal tract at colorectal cancer diagnosis. Endoscopic screening is particularly important with increasing age, history of alcohol consumption, and poor cancer differentiation.

## 1. Introduction

Based on the National Cancer Registration Annual Report 2010, in Korea, gastric cancer and colorectal cancer are the second and third most common cancers, respectively, nationwide. The incidence of colorectal cancer, in particular, is dramatically increasing owing to its association with environmental factors such as the Westernized eating habits and genetic predispositions [1]. The molecular biological pathogenesis of sporadic colorectal carcinoma, which includes most cases of colorectal cancer, involves activation of APC/*β*-catenin pathway, chromosomal instability of various oncogenes such as* p53, DCC*, and* SMAD2/4*, and microsatellite instability due to defective DNA mismatch repair gene and serrated pathways [2]. However, the pathogenesis of gastric cancer differs from that of colorectal cancer in that it is affected by racial, regional, and environmental factors and that its molecular biological pathogenesis involves genetic and epigenetic alteration and histological differentiation; all this leads to various findings within tumors or tumor heterogeneity, suggesting biologically and/or genetically heterogeneous complexity [3]. Considering the various pathogenetic mechanisms underlying colorectal and gastric cancer, it is challenging to identify the mechanism of association between the two cancers. According to some studies, among patients with positive stool occult blood test but negative of any lesions during colonoscopy, some had upper gastric lesions during upper gastrointestinal (GI) endoscopy [4–6]. Some reports have found a high correlation between upper GI lesions in patients with colorectal cancer and colon polyp [7]. These studies have classified upper GI lesions such as gastric ulcer, duodenal ulcers, gastric polyp, and gastric cancer as meaningful findings. However, only a few studies detail the frequency of precancerous lesions such as gastric adenomas or gastric cancer in colorectal cancer patients. Furthermore, the subgroup of patients who would benefit from follow-up upper GI endoscopy is not defined. Therefore, the authors investigated the difference in the frequency of occurrence of gastric adenomas or gastric cancer and the clinicopathological characteristics that affect their incidence in colorectal cancer patients.

## 2. Materials and Method

### 2.1. Materials

**Figure 1:**
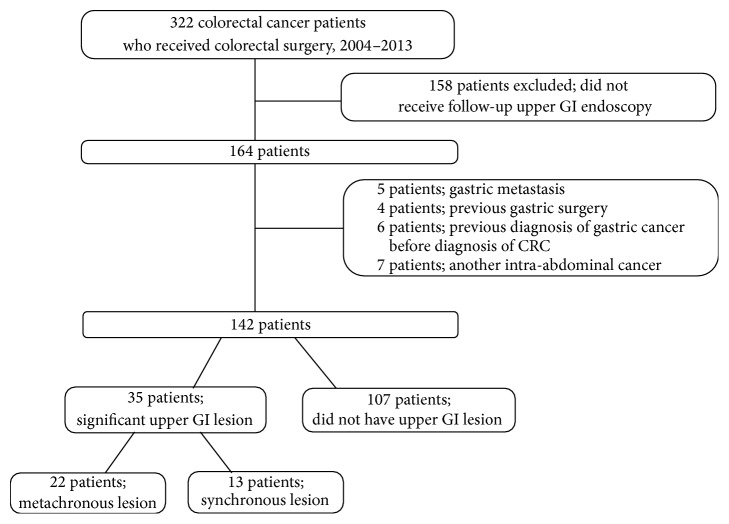
Study design and enrollment of patients. GI, gastrointestinal; CRC, colorectal cancer.

Between January 2004 and December 2013, 322 patients underwent colorectal surgery at Chungnam National University Hospital. Of these, the following patients were excluded: 158 patients who did not undergo follow-up upper GI endoscopy; 5 patients with gastric metastasis; 4 patients with previous gastric surgery, 6 patients diagnosed with gastric cancer before the diagnosis of colorectal cancer, and 7 patients with intra-abdominal cancer other than of gastric or colorectal origin. The remaining 142 patients were retrospectively studied (Figure 1). Patients with genetic diseases such as hereditary nonpolyposis colorectal cancer (HNPCC) and familial adenomatous polyposis (FAP) were also excluded for their strong association with the development of gastric cancer. We selected age, sex-matched controls from among those who were negative of colorectal cancer and rectal polyp during health screening. All patients in the matched cohort underwent follow-up upper GI endoscopy. Control group were 3 : 1 matched to patient group, and 426 patients were selected for matched control group. Colonoscopy was performed in patients who were tested from the rectum to cecum and were found to be free of rectal cancer or rectal polyp.

### 2.2. Method

Patients who were diagnosed with colorectal cancer and had undergone surgical treatment underwent upper GI endoscopy regardless of their upper GI symptoms. Precancerous lesions such as gastric adenomas or gastric cancer were regarded as significant lesions: their incidence was analyzed and their clinicopathological correlation was investigated by comparing with colorectal cancer patients negative for upper GI lesions. The colorectal cancer group was subdivided into two groups according to the presence of associated significant upper GI lesions, and the clinical characteristics were compared between the groups. In addition, among the patients with significant upper GI lesions, those whose upper GI lesions were diagnosed 6 months after the initial diagnosis of colorectal cancer (metachronous) were compared with patients without associated lesions. Demographic data included age; sex; body weight; body mass index (BMI); hypertension; diabetes; smoking history; alcohol history; family history of solid tumors; size, location, stage, histological differentiation, vessel invasion, and lymph node invasion of colorectal cancer; and carcinoembryonic antigen (CEA) levels. And, the cumulative incidence of gastric adenoma and gastric cancer was compared between the control group and colorectal cancer group. Furthermore, incidences of other upper GI lesions were compared between the two groups both at the time of diagnosis and follow-up evaluation.

### 2.3. Definition

Significant lesions were defined as precancerous lesions such as gastric adenomas or gastric cancer, and TNM stage was determined according to the American Joint Committee on Cancer (AJCC) cancer staging, seventh edition. Family history of solid tumors was defined as solid tumors occurring in the same family for two generations. Solid tumors included cancer of the colorectum, stomach, pancreas, biliary tree, esophagus, lung, larynx, and brain. Hepatocellular cancer and head and neck cancer were excluded because they are mostly of viral origin. Follow-up period was defined from the time of diagnosis of colorectal cancer in colorectal cancer group and from the time of initial health screening upper GI endoscopy for the control group, up to the end of the last upper GI endoscopy for both groups. For colorectal cancer patients, alcohol and smoking history were based upon admission records and nurse records, whereas for the control group, patients were asked to fill in a questionnaire. Both current and ex-smokers were counted as patients with smoking experience. Synchronous and metachronous lesions were classified based on the time the upper GI lesions were detected. If gastric cancer and colorectal cancer were diagnosed at the same time or within 6 months of follow-up, these lesions were defined as synchronous lesions. If the diagnosis was made after 6 months, they were defined as metachronous lesion.

### 2.4. Statistical Analysis

Based on the development of upper GI lesions in the colorectal cancer group, clinical and pathological continuous variables were analyzed using independent *t*-test, whereas categorical data analysis was conducted using a chi-square test. Multivariate analysis was done using binary logistic regression analysis. Only age was divided into five categories (under 40, 40–49, 50–59, 60–69, and over 70) and analyzed after assigning a serial number. When comparing the occurrence rate of upper GI lesions and other variables between the colorectal cancer group and the control group, independent *t*-test and chi-square test were used. The time-dependent cumulative incidence of gastric adenoma and gastric cancer between the two groups was analyzed using the Kaplan-Meyer survival analysis. Statistical calculation was performed with PASW Statistics version 18.0 (IBM Co., Armonk, NY, USA), and *p* < 0.05 was regarded as statistically significant.

## 3. Results

### 3.1. Factors Significantly Related to the Development of Upper GI Lesions in Colorectal Cancer Patients

Clinical and pathological variables were compared between 35 patients who developed significant upper GI lesions, which were detected during follow-up upper GI endoscopy after colorectal cancer surgery (13 synchronous lesions and 22 metachronous lesions), and 107 patients who did not develop such lesions. Age; sex; body weight; BMI; hypertension; diabetes; smoking, drinking, and family history of solid tumors; size, location, stage, histological differentiation, vessel invasion, and lymph node invasion of cancer; and CEA levels were the variables analyzed. Factors that were statistically significantly different between the groups were age, drinking, and lymph node invasion. When we compared the group with metachronous lesions and the group without lesions, drinking was the only factor to show statistical significance (Table 1). Multivariate analysis was performed to exclude the confounder effect: comparison of the group with both synchronous and metachronous lesions and the group with only metachronous lesions showed that age, drinking, and histological differentiation were statistically significant. Drinking and histological differentiation increased cancer risks up to approximately 6.5 and 5.9 times, respectively (Table 2).

### 3.2. Upper GI Endoscopy Findings and Their Relation to Diagnosis of Colorectal Cancer in the Colorectal Cancer Group and Control Group

The occurrence rate of gastric adenoma and gastric cancer during upper GI endoscopy at the diagnosis of colorectal cancer in the colorectal cancer group was 9.1% (13 patients) and 2.1% (9 patients) higher than that in the control group. There was no between-group significant difference in other endoscopic findings, including chronic atrophic gastritis (56.3% versus 62.7%, *p* = 0.180), gastric ulcer (1.4% versus 0.9%, *p* = 0.636), duodenal ulcer (2.1% versus 1.9%, *p* = 0.860), and hyperplastic polyp (1.4% versus 4.7%, *p* = 0.079). We however found an increased incidence of intestinal metaplasia in the colorectal cancer group when compared with the control group (10.6% versus 5.6%, *p* = 0.044; Table 3).

### 3.3. Follow-Up of Upper GI Endoscopy Findings in the Colorectal Cancer Group and Control Group

Excluded from the study were 13 patients who were diagnosed with upper GI lesions within 6 months of colorectal cancer diagnosis in colorectal cancer group and 9 patients who were diagnosed with upper GI lesions within 6 months of their first health inspection in the control group. The remaining 129 patients and 417 patients were compared for the occurrence rate of gastric adenoma and gastric cancer: the colorectal cancer group showed a higher rate, with 22 patients (17.1%) and 11 patients (2.6%), respectively (*p* < 0.001; Table 4). So, among the 35 patients who developed significant upper GI lesions in the colorectal cancer group, 13 patients (9.1%) had synchronous lesions and 22 patients (15.5%) had metachronous lesions. We also found a trend toward increased incidence of intestinal metaplasia in the colorectal cancer group when compared with the control group over time (24.8% versus 13.4%, *p* = 0.002).

### 3.4. Cumulative Occurrence of Gastric Adenoma and Gastric Cancer according to Time in Colorectal Cancer Patients

**Figure 2:**
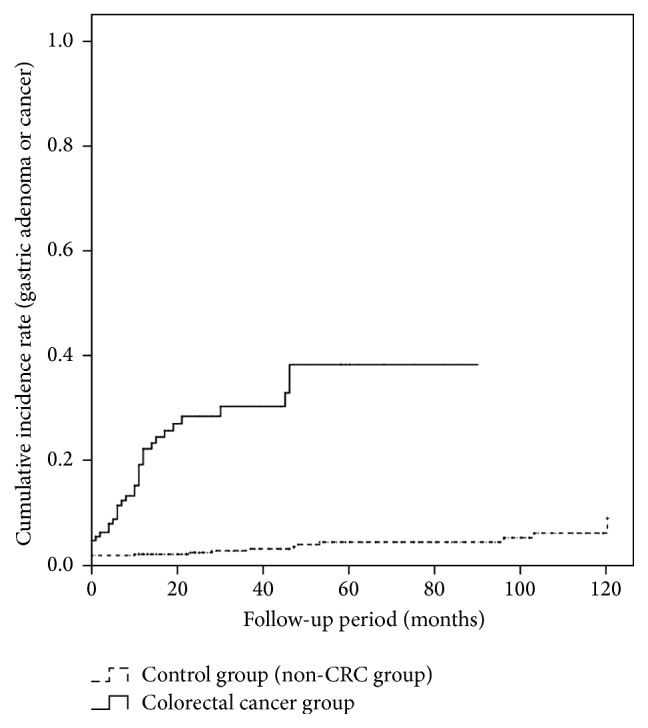
Cumulative Incidence Rate of Gastric Adenoma or Cancer in Colorectal Cancer Patients Group and Control Group. The cumulative incidence rate was higher in the colorectal cancer patients group than in the control group over the follow-up period (*p* < 0.001). Gastric adenoma or cancer mostly developed during the first 2 years of follow-up and as late as 4 years after diagnosis.

Precancerous lesions such as gastric adenoma or gastric cancer were diagnosed through follow-up upper GI endoscopy mostly within 2 years of colorectal cancer diagnosis and up to 4 years after diagnosis (Figure 2). Also, the patient group showed a significantly high accumulative incidence rate when compared with the control group (*p* < 0.001).

## 4. Discussion

Geller et al., in their study, showed that patients with colon polyp and positive stool occult blood test had a higher incidence of upper GI lesions [5]. Shin et al. showed that patients with colorectal cancer or colon polyp had a higher incidence of upper GI lesions [7]. Our results are similar to these reports in that the colorectal cancer group showed a statistically significant higher incidence of upper GI lesions when compared with the control group. It must be noted that previous studies did not restrict study patients to those with colorectal cancer; these studies included patients with benign lesions (gastric ulcer and duodenal ulcer), precancerous lesions such as gastric adenoma, and gastric cancer [4–7]. However, we studied only colorectal cancer patients and focused on the development of precancerous lesions such as gastric adenoma and gastric cancer at the time of colorectal cancer diagnosis or during follow-up.

To compare the incidence of gastric adenoma and gastric cancer between the colorectal cancer group and control group, we first compared baseline clinical characteristics and endoscopic findings. Underlying characteristics associated with the development of gastric cancer, such as age and smoking history, were not significantly different between the two groups. Body weight and diabetes were statistically significantly different between the two groups, but these factors are not known risk factors of gastric cancer. Therefore, it is difficult to say that these factors contribute to gastric cancer and gastric adenoma in the patient group. Our study, however, did not include the known risk factors of gastric cancer, such as* Helicobacter pylori* infection, patients' socioeconomic status, and blood type A, thereby limiting statistical results. Furthermore, cases of family history of solid tumors were more in the control group; this observation can be attributed solely to the data collection method: from past medical nurses' records for the patient group and survey before health inspection for the control group. Another important finding during upper GI endoscopy, other than gastric adenoma or gastric cancer, is the prevalence of intestinal metaplasia, which was higher in the colorectal cancer group than in the control group [8]. This finding is in line with the results that gastric adenoma and gastric cancer frequently occur in intestinal metaplasia, probably through a morphological factor.

Bok et al. compared the clinical and pathological characteristics of colorectal cancer accompanied with gastric cancer and of colorectal cancer alone; they found that increased age, low BMI, and peritoneal seeding of metastatic colorectal cancer were highly related [9]. However, among patients with concomitant gastric cancer and colon cancer, as 67% of patients had gastric cancer before colon cancer, most cases of colon cancer were diagnosed at follow-up examinations for gastric cancer. Also, a previous study assessed the development of colon adenoma or colorectal cancer in patients with gastric cancer. Some studies have investigated the need for screening colonoscopy in gastric cancer patients, but there is no concrete evidence favoring follow-up upper GI endoscopy in colorectal cancer patients. However, in the present study, we assessed the incidence of precancerous lesions such as gastric adenoma or gastric cancer through follow-up upper GI endoscopy in colorectal cancer patients and analyzed the contributing factors.

For colon cancer patients who had synchronous or metachronous lesion at follow-up upper GI endoscopy, we conducted multivariate analysis using two methods. First, we included all patients with synchronous and metachronous lesion, and, second, we only included patients with metachronous lesion. In both the analyses, patients with a significant upper GI lesion were older, had a history of alcohol drinking, and had poor histological differentiation of colorectal cancer when compared with patients without lesions. In this study we did not evaluate the linear association between amount of alcohol intake and significant upper GI lesion incidence. However, previous metaanalysis study shows that there is a positive association between heavy alcohol drinking (>4 drinks per day) and gastric cancer [10]. But, heavy alcohol drinking is commonly associated with poor nutrition and this could increase the risk in heavy drinkers. So, a confounding effect due to dietary habits cannot be ruled out. And, it was not factors such as staging, vascular invasion, lymphatic infiltration of colorectal cancer, and CEA levels, but histological differentiation that affected the occurrence of gastric adenoma and gastric cancer in colorectal cancer patients. In 2010, Yoon et al. studied factors related to the occurrence of synchronous or metachronous gastric cancer in colorectal cancer patients through multivariate analysis; they found that old age, male sex, family history of solid tumors, and loss of* MSH2* expression were significantly related. On the other hand, multivariate analysis showed no statistical significance of histological differentiation, a finding different from ours [11]. This difference is possibly due to the elimination of precancerous lesions such as gastric adenoma and sole inclusion of gastric cancer.

In the colorectal cancer group, when comparing the time-dependent cumulative occurrence of gastric adenoma or gastric cancer, most lesions developed within 2 years and up to 4 years. Previous studies reported similar results in that most gastric cancer and colorectal cancer appeared within 3 years [12, 13]. Also, compared with the control group, the cumulative occurrence rate was continuously high at the time of colorectal cancer diagnosis and even during the entire follow-up period after colorectal cancer surgery [14–19]. This shows that genetic factors are also as important as morphological factors in the pathogenesis of gastric cancer and colorectal cancer. Gastric cancer and colorectal cancer possess identical genetic changes in* p53, APC, DCC*, and* K-ras* [14–17]. Genes such as* hMLH1* and* hMSH2*, which play an important role in HNPCC, appear in 10% to 15% of sporadic gastric cancer and colorectal cancer cases [18]. Microsatellite instability (MSI) is reported to appear in 18% of solitary gastric cancer cases, and in 13% to 17% of solitary colorectal cancer cases [19], suggesting, though partially, a genetic causality.

Our study has several limitations. First, this is a retrospective analysis of past medical records of a single center. Second, data on alcohol consumption was collected solely from past medical records; therefore, exact amount or duration of alcohol consumption remains uncertain. Lastly, not all patients underwent annual follow-up upper GI endoscopy during the study period.

Our results show higher occurrence rates of precancerous lesions such as gastric adenoma and gastric cancer in colorectal patients than in the general population group. In patients with colorectal cancer, increased age, alcohol history, and poor histologic differentiation were associated with higher incidence of gastric adenoma and gastric cancer in overall patients with significant upper GI lesions, and such clinical correlation is also observed in the patients with metachronous lesions alone. Also, follow-up upper GI endoscopy revealed that most lesions appeared within 2 years and up to 4 years.

## 5. Conclusions

In comparison to previous studies, our results suggest that screening with upper GI endoscopy at the time of colorectal cancer diagnosis is very important, especially with factors such as increased age, history of drinking, and poor histological differentiation. It is also important to note that even if lesions such as gastric adenoma or gastric cancer are not present at the time of diagnosis in colorectal cancer patients with these risk factors, there is a high possibility of its development in the future. Therefore, it is important to perform follow-up upper GI endoscopy screening, even 4 to 5 years after colorectal cancer surgery.

Finally, further research is needed to reveal the interactive association between the development of colorectal cancer and gastric cancer, and studies on molecular biologic interactive mechanisms and larger prospective studies are needed.

## Figures and Tables

**Table 1:** Comparison of clinicopathologic features in the subjects with or without UGI lesion in colorectal cancer patients.

Variables	Patients without UGI lesion (*n* = 107)	Patients with synchronous and metachronous UGI^a^ lesion (*n* = 35)	*p* value	Patients with metachronous UGI^a^ lesion (*n* = 22)	*p* value
Sex			0.078		0.060
Male	65 (60.7%)	27 (77.1%)		18 (81.8%)	
Female	42 (39.3%)	8 (22.9%)		4 (18.2%)	
Age (yrs)	64.76 ± 11.72	68.29 ± 7.90	0.048	68.36 ± 8.01	0.128
Body weight (kg)	58.50 ± 9.26	60.78 ± 9.09	0.918	61.91 ± 9.95	0.650
BMI (body mass index, kg/m^2^)	22.60 ± 3.05	23.01 ± 2.97	0.723	23.44 ± 3.13	0.931
Hypertension	33 (30.8%)	12 (34.3%)	0.704	8 (36.4%)	0.612
Diabetes	19 (17.8%)	10 (40.0%)	0.168	6 (27.3%)	0.304
Smoking	21 (19.6%)	12 (34.3%)	0.075	7 (31.8%)	0.206
Alcohol	25 (26.4%)	20 (57.1%)	<0.001	12 (54.5%)	0.003
Family history (solid tumor)	10 (9.35%)	6 (17.1%)	0.205	4 (18.2%)	0.225
Tumor size (cm)^b^	5.06 ± 2.39	4.49 ± 3.02	0.504	4.57 ± 3.46	0.271
CRC location^c^			0.828		0.844
Rt.colon	62 (57.9%)	21 (60.0%)		14 (63.6%)	
Transverse colon	4 (3.7%)	2 (5.7%)		1 (4.6%)	
Lt.colon	41 (38.3%)	12 (34.3%)		7 (31.8%)	
Cancer staging			0.749		0.907
I	22 (20.6%)	10 (28.6%)		6 (27.3%)	
II	42 (39.3%)	11 (31.4%)		8 (36.4%)	
III	36 (33.6%)	12 (34.3%)		7 (31.8%)	
IV	7 (6.5%)	2 (5.7%)		1 (4.5%)	
Vascular invasion	84 (78.5%)	22 (62.9%)	0.065	15 (68.2%)	0.297
Lymphatic invasion	86 (80.4%)	21 (60.0%)	0.015	14 (63.6%)	0.087
Differentiation			0.077		0.147
Well differentiated	2 (1.9%)	1 (2.86%)		0 (0%)	
Moderate differentiated	101 (94.4%)	29 (82.9%)		19 (86.4%)	
Poorly differentiated	4 (3.7%)	5 (14.3%)		3 (13.6%)	
CEA level (ng/mL)	8.20 ± 18.71	6.22 ± 12.31	0.530	5.58 ± 11.20	0.417
Follow-up duration (month)	25.57 ± 23.75	36.69 ± 27.55	0.581	29.77 ± 22.4	0.417

^a^Upper GI lesion includes gastric adenoma or cancer.

^b^Length of the long axis of the tumor.

^c^Tumors located in the cecum and ascending colon were categorized as Rt.colon and those located in descending colon and S-colon, rectum, were categorized as Lt.colon.

**Table 2:** Multivariate analysis for the clinicopathologic factors associated with gastric adenoma or gastric cancer in colorectal cancer patients.

Variables	Group with both synchronous and metachronous lesions			Group with only metachronous lesions		
	Odds ratio	*p* value	95% CI	Odds ratio	*p* value	95% CI
Age	1.062	0.015	1.012–1.116	1.885	0.048	1.005–3.534
BMI	1.066	0.417	0.913–1.244	1.124	0.209	0.936–1.351
Smoking	1.521	0.442	0.523–4.425	1.427	0.577	0.409–4.972
Alcohol	6.506	<0.001	2.388–17.728	6.314	0.002	1.952–20.430
Family history (solid tumor)	3.201	0.065	0.930–11.020	3.797	0.076	0.870–16.578
Differentiation	5.901	0.029	1.202–28.970	9.748	0.022	1.390–68.366
Vascular invasion	1.027	0.965	0.303–3.484	1.252	0.759	0.299–5.236
Lymphatic invasion	0.424	0.174	0.123–1.461	0.528	0.385	0.125–2.228

**Table 3:** Comparison of baseline characteristics and endoscopic findings in the subjects with colorectal cancer patients and control group.

Variables	Colorectal cancer patients (*n* = 142)	Control group (*n* = 426)	*p* value
Sex			0.105
Male	91 (64.1%)	240 (56.3%)	
Female	51 (35.9%)	186 (43.7%)	
Age (yrs)	65.63 ± 10.98	61.87 ± 9.99	0.184
Body weight (kg)	59.06 ± 9.23	64.67 ± 11.02	0.023
BMI (body mass index, kg/m^2^)	22.71 ± 3.03	23.97 ± 3.29	0.583
Hypertension	45 (31.7%)	110 (25.8%)	0.174
Diabetes	29 (20.4%)	55 (12.9%)	0.029
Smoking	33 (23.2%)	71 (16.7%)	0.079
Alcohol	45 (31.7%)	162 (38.0%)	0.174
Family history (solid tumor)	16 (11.3%)	103 (24.2%)	0.001
Follow-up period (months)	28.3 ± 25.11	44.4 ± 25.37	0.577
Baseline endoscopic findings			
Chronic atrophic gastritis	80 (56.3%)	267 (62.7%)	0.180
Intestinal metaplasia	15 (10.6%)	24 (5.6%)	0.044
Gastric ulcer	2 (1.4%)	4 (0.9%)	0.636
Duodenal ulcer	3 (2.1%)	8 (1.9%)	0.860
Hyperplastic polyp	2 (1.4%)	20 (4.7%)	0.079
Gastric adenoma	3 (2.1%)	7 (1.6%)	0.713
Gastric cancer	10 (7.0%)	2 (0.5%)	<0.001
Subepithelial tumor (SET)	3 (2.1%)	0 (0%)	0.003

**Table 4:** Follow-up endoscopic findings in colorectal cancer group and control group.

Endoscopic findings	Colorectal cancer patients (*n* = 129)	Control group (*n* = 417)	*p* value
Chronic atrophic gastritis	86 (66.7%)	289 (69.3%)	0.572
Intestinal metaplasia	32 (24.8%)	56 (13.4%)	0.002
Gastric ulcer	7 (5.4%)	11 (2.6%)	0.121
Duodenal ulcer	7 (5.4%)	11 (2.6%)	0.121
Hyperplastic polyp	5 (3.9%)	35 (8.4%)	0.085
Gastric adenoma	9 (7.0%)	9 (2.2%)	0.007
Gastric cancer	13 (10.0%)	2 (0.5%)	<0.001
Subepithelial tumor (SET)	3 (2.3%)	0 (0%)	0.002
